# Validation of Five Facets Mindfulness Questionnaire – Short form, in Spanish, general health care services patients sample: Prediction of depression through mindfulness scale

**DOI:** 10.1371/journal.pone.0214503

**Published:** 2019-04-02

**Authors:** Ángela Asensio-Martínez, Barbara Masluk, Jesus Montero-Marin, Bárbara Olivan-Blázquez, Maria Teresa Navarro-Gil, Javier García-Campayo, Rosa Magallón-Botaya

**Affiliations:** 1 Research Network in preventive activities and health promotion RedIAPP, Barcelona, Spain; 2 Health Research Institute of Aragon, Zaragoza, Spain; 3 Department of Psychology and Sociology, University of Zaragoza, Zaragoza, Spain; 4 Department of Medicine, Psychiatry and Dermatology, University of Zaragoza, Zaragoza, Spain; Universita degli Studi Europea di Roma, ITALY

## Abstract

The validation of Five Facets Mindfulness Questionnaire (FFMQ)—short version was performed in a general population of a city in a region of Galicia (Spain), the sample was composed of randomly selected Spanish Health Care patients (N = 845). The results on the goodness of fit of the non-hierarchical, five-dimensional factorial model met the criteria for good and acceptable model adjustment (after eliminating item 18 and despite the correlations detected among the errors included in the model), explaining a 55.5%.of the variance. As the second objective has been analysed the association between the scores obtained in the different facets of the FFMQ-SF and the risk of suffering an episode of depression. (The Odd Ratio, the Hosmer-Lemeshow test and the ROC curve were calculated.) Participants who were currently suffering from an episode of depression were more likely to have low scores in "describing" facet of Mindfulness (adjusted OR = 1.58, 95% CI = 1.04–2.40, linear trend: χ2 = 3.74, df = 1, p = 0.053) as well as low scores on "acting with awareness" (adjusted OR = 2.19, 95% CI = 1.461–3.30, linear trend: χ2 = 9.52, df = 1; = 0.002) and "non judging" (adjusted OR = 2.05, 95% CI = 1.36–3.09, linear trend: χ2 = 143.21, df = 1; p <0.001). Participants with a previous episode of depression were more likely to have low scores on the subscale "acting with awareness" (adjusted OR = 2.37, 95% CI = 1.43–3.93, linear trend: χ2 = 9.62, df = 1, p = 0.002) and "non-reactivity" (adjusted OR = 2.14, 95% CI = 1.28–3.56, linear trend: χ2 = 8.30; df = 1; p = 0.004. Questionnaire FFMQ-SF is an adequate questionnaire for the evaluation of mindfulness in non-clinical multi- occupational population.

## Introduction

The concept of Mindfulness is the English translation of the term "sati" of the Pali language (language of Buddhist scriptures), which carries the meaning of attention or being conscious, and being present [1]. Several authors propose different components of mindfulness, but all of them start from the proposal of factors of Jon Kabat-Zinn, 1990 [2], which raises seven factors related to the attitude that constitute the main supports of mindfulness: non judging, patience, beginner's mind, trust, non-striving, acceptance and letting go. In current psychology, mindfulness can be considered as a psychological resource to increase awareness and skillfully responding to mental processes that contribute to psycho-emotional stress and poor adaptive functioning [3].

In terms of differentiating mindfulness from other cognitive processes such as intelligence or skill, Sternberg considers that despite a possible overlap with these cognitive processes, mindfulness possesses features more typical of cognitive style. [4].

In terms of biological-neuronal functioning, mindfulness is considered a trainable construct and its training is associated with the regulation of emotions "top-down" in the short term practitioners, and with 'bottom-up' in the long term practioners [5]. Hence, in the absence of consensus about the construction there are several questionnaires that measure mindfulness considered as a multifaceted construct (scales that contain subscales that measure their dimensions separately), or a one-dimensional construct (unitary scale). Likewise, it can be measured as a trait or as a psychological state.

There is ample scientific evidence that cnfirms the effectiveness of different therapies based on mindfulness for the improvement of different aspects of health and physical and mental illnesses. It has been studied in pathologies such as chronic pain [6–8], fibromyalgia [9–12], cancer [13–18], HIV [19,20], cardiovascular diseases [21], transplant patients [22], tension headache [23], and asthma [24]. Regarding its efficacy in mental illnesses, it has been evidenced in investigations in patients with depression [25–30], and especially prevention of recurrence, anxiety [31–35], post-traumatic stress disorder [36,37], obsessive-compulsive disorder [38], eating disorders [39–42] and substance abuse [43,44].

However, as some researchers point out [45], the increase in interest in mindfulness as a useful technique in the treatment of mental illness, substance abuse and others, is not linked to an increase in the methodological quality of the studies. In their recent study Goldberg and colleagues indicate that the conclusions of the effectiveness of mindfulness can be formulated based on few high quality studies. We can also expect that many of the treatments that have been carried out have had a preliminary character and that therefore in the next few years trials will be carried out with a more strict methodology.

Sauer et al. [46], carried out a review of the state of the issues in the measurement of mindfulness. They concluded that the scales predominantly used are the Kentucky Inventory of Mindfulness Skills (KIMS) and the Mindful Attention Awareness Scale (MAAS), and recommend the use of the Mindful Attention Awareness Scale as it has been shown to be useful in a large number of studies. For more in-depth analysis, including the possibility of investigating different facets of mindfulness, the Five Facets Mindfulness Questionnaire is recommended by the authors; since it incorporates existing instruments such as KIMS and differentiates several subcomponents of mindfulness.

The Five Facets Mindfulness Questionnaire (FFMQ) [47] is a scale of 39 items, developed by a part of the authors of the scale KIMS, Baer and collaborators. It arises from a factorial analysis of the five main scales that measure mindfulness: KIMS, Freiburg Mindfulness Inventory (FMI), MAAS, Cognitive and Affective Mindfulness Scale (CAMS) and Southampton Mindfulness Questionnaire (SMQ). The authors concluded that within the construct of mindfulness there would be five factors: observing, describing, acting with awareness, non- judging internal experience and non- reactivity to internal experience. The scale presents positively and negatively worded items, and a differential response according to the respondent population: meditators or non-meditators; there is a greater sensitivity to this in non-meditators when they score positively worded (full attention capacity) or negatively worded (attention deficit) items [48]. The authors recommend not using the total score and that the values of the subscales should be used. Both in its original version and in its Spanish version it is a reliable and valid instrument to evaluate the different aspects of mindfulness[48]. With the purpose of saving instabilities with respect to their dimensions, the redundancy of items and the differential functioning of the same, several short versions of the questionnaire were developed: a short and reliable Dutch version of the FFMQ (FFMQ-SHORT FORM) of 24 items [49], German short version (FFMQ-SHORT) of 20 items validated in Austrian subjects [50] and the Chinese brief version of 20 items [51]. The FFMQ-SHORT FORM (FFMQ-SF) does not present a Spanish validation in non-clinical cross-sectional population.

There is a need for validation in the general population, of one of the most used questionnaires for measuring the psychological construct of mindfulness: the FFMQ-SF. This questionnaire has been previously validated in its long version in clinical and non-clinical Spanish population, but the short version has not been validated until now. The short version of the FFMQ-SF questionnaire will offer the advantages over the long version as it can be administered more easily compared to the long version.

Therefore, the objective of the present study was to validate the FFMQ-SF questionnaire in the general population. It is expected to present good psychometric qualities of reliability and validity in its five component dimensions, in a general multi-occupational population. Given the effectiveness of the Mindfulness-based interventions in depression and especially in the prevention of relapses, and given the high prevalence of this psychopathology [52,53], a second objective was proposed to analyze the relationship between the scores obtained in the different facets of the FFMQ-SF and risk of suffering a previous or current episode of depression.

## Methodology

### Design of the study

A descriptive and analytical study has been carried out with cross-sectional sample.

### Sample and sample size

The study was developed in the municipality of A Estrada, Galicia (Spain), whose adult population (18 years and older) consists of 18,879 inhabitants in 2012. The inclusion criteria were: 1) Population over 18 years of age; 2) Proficiency in spoken Spanish or ability to communicate; 3) Grant informed consent. The exclusion criteria were: 1) Presence of severe chronic disease (dementia, mental retardation, cerebrovascular disease, terminal cancer, etc.).

The sample size needed for each construct was calculated to make inference. Based on the objectives pursued, assuming for the population studied, with a confidence level of 95% and a precision of 2, for resilience, according to the study by Beutel et al.[54], the most unfavorable standard deviation is 10.68 so 110 patients were needed to ensure the general health variable; according to Terry [55], the standard deviation is 4.98 and for self-compassion it is 3.96, so 24 and 16 patients were needed; for mindfulness, according to the study by Carmody et al [56]. The most unfavorable standard deviation is 14.87, which would require 202 patients.

Considering the larger sample size needed to make inference, taking into account that the percentage of participation and eligibility based on previous studies from a conservative perspective, the sample was increased to 845 patients. These patients were randomly selected based on the Health Care Registry. A computer program ("sample" function of cran-R.org) generated a random sample of equal numbers of subjects stratified by age groups (in 7 categories, each one for every ten years).

Of the initial sample selected from 3500 subjects, it was not possible to contact 639, 134 subjects resided outside A Estrada, 19 had no right to health care, and 84 had died. Of the remaining eligible patients (n = 2624), 394 subjects were excluded from the study because they did not meet the inclusion criteria. The reasons for exclusion were cognitive impairment, immobilization, walking problems, active neoplasia, addictions, terminal illnesses, cerebrovascular accident, serious psychiatric illness, social disorder, pregnancy, Parkinson's and mainly sensory problems. 714 patients have refused to participate in the study and 1516 patients signed the informed consent. 845 participants have completed the questionnaire.

### Instruments

To validate the Five Facets of Mindfulness Questionnaire (FFMQ-SF) in a multi- occupational sample, as a starting point, we chose the 24-item questionnaire of the Dutch short version, validated in the clinical population (anxious and depressive symptomatology), as it is a European sample and present good psychometric properties (α> 0.70 in all dimensions) [49], obtaining the translation of the items directly from the original version of the FFMQ validated in Spanish by Cebolla et al. [48].

This questionnaire assesses the ability of the subject to be aware in the experience of the moment. The items are answered on a Likert scale ranging from 1 (*never* or *very rarely true*) to 5 (*very often* or *always true*). It contains positively worded items (capacity for mindfulness) and others negatively worded (attention deficit) It evaluates five facets of mindfulness [49]:

1. **Observing:** realize internal and external experiences such as sensations, emotions and thoughts (items 6, 10, 15, 20: "I pay attention to the sensations produced by the wind in the hair or the sun on the face").
2. **Describing:** label internal experiences with words (items 1, 2, 5, 11, 16: "I am good at finding the words to describe my feelings")
3. **Acting with awareness:** focusing on one’s activities in the moment as opposed to behaving mechanically (items 8, 12, 17, 22, 23: "It is difficult for me to stay focused on what is happening in the present").
4. **Non judging internal experience:** refers to taking a non-evaluative stance toward thoughts and feelings (Items 4, 7, 14, 19, 24: "I tell myself that I should not feel what I feel")
5. **Non reactivity to internal experience:** allowing the free flow of thoughts and emotions without getting caught up in by them or without rejecting them (items 3, 9, 13, 18, 21: "I observe my feelings without getting lost in them").

A score of the general scale is obtained by adding the value of all the items, from 39 to 195, taking into account the presence of items that are scored in an inverse manner (4, 5, 7, 8, 11, 12, 14, 17, 19, 22, 23, 24), and an individual score on each subscale from 5 to 25, except for the "observe" dimension that ranges from 4 to 20. Higher scores indicate a greater capacity for mindfulness.

To analyze the associations between the scores obtained in the different facets of the FFMQ-SF and risk of having a current or past episode of depression, the Goldberg Anxiety and Depression Scale was administered to the entire sample: symptoms of anxiety and depression in the sample has been used the scale of anxiety and depression of Goldberg in its version validated in Spain by Montón et al. [57], which presents a sensitivity of 83.1% and a specificity of 81.8%. The subscale of depression shows a high sensitivity to capture patients diagnosed with depressive disorders (85.7%), and the subscale of anxiety has a somewhat lower sensitivity (72%). These are two self-administered scales of 9 items each (eg, "Have you felt very excited, nervous or tense?", "Have you been very worried about something?", "Have you felt with little energy?","Have you lost interest in things?"), of which the first four are mandatory, and the remaining five elements are only administered if a certain number of positive responses is reached on each scale: one answer on the depression scale and two answers on the anxiety scale. The response options for each item are reduced to Yes or No. Scores above 2 on the depression scale and above 4 on the anxiety scale are considered probable cases of depression and anxiety, respectively. The scale was designed as an interview guide for the doctor in daily clinical practice, to know the existence of psychopathological disorder, the diagnostic orientation and its severity, and as an indicator of the prevalence of anxiety and depression disorders in a given population [57].

### Procedure

The sample was recruited from November 2012 to March 2015. All randomly selected subjects were contacted by postal mail first and then by telephone in order to explain the study and summon them to the health center. In case of agreeing to participate, they were assigned an appointment in which a trained research assistant explained the study, obtained the consent, solved doubts and collected the data.

The participants were asked to complete the questionnaire. If case of any doubt, the medical history was consulted. Each patient was assigned an anonymous identification number, thus maintaining the confidentiality of the data collected.

#### Statistical analysis

To validate the FFMQ-SF questionnaire in the general, non-clinical, multi- occupational population, the 5-dimensional model described previously was proposed [46]. We calculated means (standard deviations), medians (interquartile intervals) and frequencies (percentages) for sociodemographic data. The asymmetry, kurtosis and Mardia coefficients [58] have been analyzed to evaluate the distribution of the items. We use polymorphic matrices to estimate a more real correlation between the theoretical continuous latent variables of the ordinal items observed, and we estimate the characteristics of the matrices by the determinant of the correlation matrix, the KMO index and the Bartlett sphericity test [59].

The determinant of the correlation matrix shows the extent to which the variables are related and is zero when they are linearly dependent. The KMO index of sampling adequacy predicts whether the data is likely to have a good factor (KMO must be ≥0.70 to continue factor analysis).

We use the goodness-of-fit index (GFI) and the adjusted goodness-of-fit index (AGFI), which refer to the explained variance of the model, and values> 0.90 are acceptable [54]; also the standardized mean square residual (RMSR), which is the standardized difference between the observed and predicted covariance, and the square root of the mean squared error (RMSEA), which indicate good adjustment values when they score <0.08 [54]; the standardized adjustment index (NFI) that measures the proportional reduction in the adjustment function when going from null to the proposed model, and the comparative adjustment index (CFI), in both is considered a good fit of the model when they are> 0.90 [60].

To analyze the relationship between the scores obtained in the different facets of the FFMQ-SF and the suffering or not, a current or past episode of depression, the Odd Ratio, the Hosmer-Lemeshow test and the ROC curve were calculated.

The data from the questionnaires were analyzed statistically with the statistical package SPSS 20 and AMOS v20. The cases that presented data misings were eliminated.

#### Ethical considerations

The study was approved by the Regional Committee of Ethics of Medical Research, Santiago de Compostela, Spain, (2012–025), and by the Research Ethics Committee of the Autonomous Community of Aragon, Spain (15/2017). The study was conducted in accordance with the Declaration of Helsinki [61]. All participants provided written informed consent and the data was anonymized.

## Results

### Study 1. Evidence validity of internal structure

Tables 1 and 2 describe the characteristics of the sample regarding different sociodemographic variables, both physical and mental health. Regarding the validation of the questionnaire, Table 3 presents the descriptive and normative data of the factors (dimensions) of the FFMQ-SF questionnaire. It can be said that the sample presents mean results in the facet of observing and non-reactivity (evaluated by the FFMQ) and medium-high values in the rest of facets of mindfulness.

**Table 1:** Sociodemographic characteristics of the sample.

Age ^a^ (range: 18–88)	49.16 (16.97)
Sex, male ^b^	386 (43.8)
**Marital status** ^b^	
Married	558 (63.3)
Widowed	63 (7.1)
Separated / divorced	58 (6.6)
Single	203 (23.0)
**Level of education** ^b^	
Without studies / formal education	146 (16.6)
Primary studies	382 (43.4)
Secondary studies	225 (25.5)
Higher education	129 (14.5)
**Employment status** ^b^	
Employee	382 (43.4)
On sick leave	15 (1.7)
Unemployed	150 (17.0)
Homemaker	65 (7.4)
Retired	212 (24.1)
Student	43 (4.9)
Other	15 (1.7)
**Employed at least for 1 year** ^b^	744 (84.4)

n = 882

^a^ Mn (SD)

^b^ Frequency (percentage)

**Table 2:** Physical and mental health characteristics of the sample.

**Physical health**		
	SF-36 ^a^^,^ ^c^	46.42 (9.74)
	Tobacco ^b^	
	no	457 (51.8)
	ex-smoker	235 (26.6)
	yes	190 (21.5)
	Alcohol ^b^	
	0–9 gr/ per week	328 (37.2)
	10–139 gr/ per week	355 (40.2)
	140–279 gr/ per week	120 (13.6)
	280+ gr/ per week	79 (9.0)
	Physical activity ^b^	
	low	328 (37.2)
	moderate	327 (37.1)
	high	227 (25.7)
**Mental health**		
	SF-36 ^a^^,^ ^c^	48.65 (11.61)
	Anxiety, yes ^b^	193 (22.0)
	Depression, yes ^b^	219 (24.9)
	Episode of depression, yes ^b^	123 (13.9)

n = 882

^a^ Mn (SD)

^b^ Frequency (percentage)

^c^ Summary standardized questionnaire component SF-36.

**Table 3:** Descriptive and normative data of the factors of the FFMQ-SF.

Factors	n	range	Mn	SD	Q_25_	Q_33_	Md	Q_66_	Q_75_
Observing*	778	4–20	13.20	3.60	11.00	12.00	13.00	15.00	16.00
Describing*	795	5–25	16.47	4.32	14.00	15.00	16.00	18.00	20.00
**Acting with awareness** *	744	5–25	20.00	3.72	18.00	19.00	20.00	22.00	23.00
Non judging*	773	5–25	16.57	4.07	14.00	15.00	17.00	18.00	19.00
**Non reactivity to internal experience**	733	4–20	12.93	3.27	11.00	12.00	13.00	14.00	15.00

* Items with the original number

n = number of observations; Range = range of possible values; Mn = mean; SD = standard deviation. Q = quartile. Md = median.

The Mardia multivariate statistic was 41.53 (p <0.001). The correlation matrix of the items of the FFMQ-SF was significant p <0.001; the KMO test had a value of 0.84; the Bartlett statistic was of 4869.3 (df = 276), p <0.001. Mainly, the burden of factors for the confirmatory factorial analysis (CFA) was adequate (Table 4), although item no. 18 ("When I have disturbing thoughts or images I am able to notice them without reacting") was not significant (w = 0.07; p = 0.129).

**Table 4:** Descriptive statistics, regression coefficients and reliability of the FFMQ-SF factors.

Factors	α	M	SD	Assymetry	Kurto-sis	SW	RW	SE	*p*
Observing*	0.65								
Item 6		3.73	1.37	0.21	-1.17	0.43			
Item 10		3.18	1.30	-0.13	-1.03	0.44	0.97	0.11	<0.001
Item 15		4.03	1.06	-0.92	0.08	0.62	1.12	0.15	<0.001
Item 20		3.25	1.40	-0.30	-1.15	0.56	1.35	0.16	<0.001
Describing*	0.79								
Item 1		3.12	1.18	-0.19	-0.71	0.86			
Item 2		3.28	1.10	-0.18	-0.66	0.83	0.90	0.04	<0.001
Item 5		3.30	1.17	-0.29	-0.72	0.49	0.56	0.05	<0.001
Item 11		3.43	1.18	-0.39	-0.66	0.42	0.48	0.05	<0.001
Item 16		3.35	1.16	-0.23	-0.74	0.62	0.71	0.04	<0.001
**Acting with awareness** *	0.80								
Item 8		3.74	1.08	-0.57	-0.31	0.55			
Item 12		4.23	0.96	-1.17	0.80	0.59	0.94	0.10	<0.001
Item 17		3.81	1.02	-0.70	0.14	0.65	1.11	0.11	<0.001
Item 22		4.08	0.97	-0.84	0.07	0.85	1.37	0.12	<0.001
Item 23		4.06	0.98	-0.89	0.25	0.84	1.38	0.12	<0.001
Non judging*	0.73								
Item 4		3.28	1.10	-0.16	-0.73	0.52			
Item 7		3.15	1.21	-0.06	-0.88	0.41	0.83	0.11	<0.001
Item 14		3.29	1.14	-0.15	-0.63	0.67	1.29	0.12	<0.001
Item 19		3.67	1.15	-0.58	-0.40	0.72	1.41	0.15	<0.001
Item 24		3.15	1.20	-0.02	-0.82	0.42	0.86	0.13	<0.001
**Non reactivity**	0.68								
Item 3		3.19	1.07	-0.07	-0.60	0.63			
Item 9		3.17	1.30	-0.19	-0.81	0.43	0.76	0.09	<0.001
Item 13		3.36	1.30	-0.33	-0.54	0.33	0.55	0.08	<0.001
Item 21		3.21	1.18	-0.27	-0.69	0.32	0.55	0.08	<0.001

* Items with the original number.

M = average. SD = standard deviation. SW = standardized coefficient. RW = regression coefficient. SE = standardized error. p = p-value associated with the regression coefficient tems with the original numbers.

Therefore, it was eliminated, which allowed to increase Cronbach's alpha values of the corresponding factor from 0.63 to 0.68 (Table 4). In general, the internal consistency ranged between 0.65 (“Observing” factor) and 0.80 (“Acting with awareness” factor), and the standardized weights of the items were adequate, all greater than 0.30 (Table 4).

The Confirmatory Factor Analysis (CFA) adjustment indexes (after eliminating item # 18) were adequate: X^2^ = 355.72, Df = 175, X^2^ / df = 2.03, CFI = 0.96, IFI = 0.96, GFI = 0.95, AGFI = 0.93, NFI = 0.93, TLI = 0.94, RMSEA = 0.04 (90% CI = 0.03–0.05), RSMR = 0.06, explaining a 55, 5% of the variance, although the modification indexes suggested some correlations between the errors, which were included in the model ‒ items 2 ‒ 21 (r = 0.34, p <0.001); items 9 ‒ 20 (r = 0.35, p<0.001); items 3 ‒ 11 (r = -0.39, p<0.001); items 12 ‒ 18 (r = 0.34, p<0.001); items 4 ‒ 9 (r = 0.32, p<0.001); items 4 ‒ 20 (r = 0.25, p<0,001); items 13 ‒ 1 (r = -0.27, p<0.001); items 3 ‒ 8 (r = 0.28, p<0,001); items 6 ‒ 14 (r = 0.20, p<0.001); items 8 ‒ 11 (r = -0.22, p<0.001); items 12 ‒ 6 (r = 0.22, p<0.001) and items 23 ‒ 20 (r = -0.22, p<0.001).

### Study 2. Evidence of the risk of suffering an episode of depression

Regarding the relationship between the scores obtained in the FFMQ-SF and the risk of a current episode of depression, Table 5 shows the adjusted ORs. As we can see, participants with low scores in "describe" were more likely to have depression (adjusted OR = 1.58, 95% CI = 1.04–2.40, linear trend: χ2 = 3.74, df = 1, p = 0.053). Participants with low scores on "acting with awareness" were more likely to have depression (adjusted OR = 2.19, 95% CI = 1.461–3.30, linear trend: χ2 = 9.52, df = 1; = 0.002). Participants with low scores on "not judging" were more likely to have depression (adjusted OR = 2.05, 95% CI = 1.36–3.09, linear trend: χ2 = 143.21, df = 1; p <0.001).

**Table 5:** Associations between FFMQ-SF factors and current symptoms of depression.

Factors	Current depressive episode (%)	No depression (%)	OR (95% CI)	p
**Age**				
<36	42 (18.3%)	188 (81.7%)	ref.	
>36	177 (80.8%)	472 (72.7%)	1.77 (1.13–2.77)	0.013
**Gender**				
male	51 (13.2%)	334 (86.8%)	ref.	
female	168 (34.0%)	326 (66.0%)	3.32 (2.18–5.05)	<0.001
**Observing**				
high	132 (25.2%)	392 (74.8%)	ref.	
low	62 (24.6%)	132 (25.2%)	0.86 (0.55–1.33)	0.491
**Describing**				
high	111 (20.6%)	429 (79.4%)	ref.	
low	86 (34.0%)	167 (66.9%)	1.58 (1.04–2.40)	0.034
**Acting with awareness**				
high	99 (19.3%)	415 (80.7%)	ref.	
low	91 (39.9%)	137 (60.1%)	2.19 (1.46–3.30)	<0.001
**Non judging**				
high	104 (19.4%)	433 (80.6%)	ref.	
low	90 (38.5%)	144 (61.5%)	2.05 (1.36–3.09)	0.001
**Non reactivity**				
high	103 (20.7%)	394 (79.3%)	ref.	
low	82 (35.0%)	152 (65.0%)	1.84 (1.21–2.82)	0.005

OR = odds ratio. 95% CI = 95% confidence interval. P = p-value related to odds ratio

No significant differences were found between the observed and expected scores when the Hosmer-Lemeshow test was applied (χ2 = 3.96, df = 8, p = 0.861). The area under the ROC curve was 0.76 (95% CI = 0.72–0.81, p <0.001).

Table 6 shows the ORs adjusted for the existence of previous episodes of depression, according to the FFMQ-SF factors. As we can see, participants with low scores on "acting with awareness" were more likely to have a previous episode of depression (adjusted OR = 2.37, 95% CI = 1.43–3.93, linear trend: χ2 = 9.62, df = 1, p = 0.002). Participants with low scores on "non-reactivity" were more likely to have a previous episode of depression (adjusted OR = 2.14, 95% CI = 1.28–3.56, linear trend: χ2 = 8.30; df = 1; p = 0.004). No significant differences were found between the observed and expected scores when the Hosmer-Lemeshow test was applied (χ2 = 5.78, df = 8, p = 0.671). The area under the ROC curve was 0.73 (95% CI = 0.67–0.79, p <0.001).

**Table 6:** Associations between the FFMQ-SHORT FORM factors and the previous episodes of depression.

Factors	Previous depresion (%)	No previous depresion (%)	OR (95% CI)	p
**Age**				
<36	15 (6.5%)	216 (93.5%)	ref.	
>36	108 (16.6%)	543 (83.4%)	3.12 (1.65–5.90)	<0.001
**Gender**				
male	27 (7.0%)	359 (93.0%)	ref.	
female	96 (19.4%)	400 (80.6%)	2.88 (1.68–4.93)	<0.001
**Observing**				
high	66 (12.5%)	460 (87.5%)	ref.	
low	37 (14.7%)	215 (85.3%)	1.08 (0.64–1.83)	0.780
**Describing**				
high	64 (11.8%)	477 (88.2%)	ref.	
low	43 (16.9%)	211 (83.1%)	1.04 (0.62–1.76)	0.885
**Acting with awareness**				
high	51 (9.9%)	463 (90.1%)	ref.	
low	49 (21.3%)	181 (78.7%)	2.37 (1.43–3.93)	0.001
**Non judging**				
high	69 (12.8%)	470 (87.2%)	ref.	
low	35 (15.0%)	199 (85.0%)	0.73 (0.43–1.26)	0.259
**Non reactivity**				
high	45 (9.1%)	452 (90.9%)	ref.	
low	50 (21.2%)	186 (78.8%)	2.14 (1.28–3.56)	0.004

OR = odds ratio. 95% CI = 95% confidence interval. P = p-value related to odds ratio.

All data are fully available on http://doi.org/10.3886/E108404V1. The authors will post the procedure used and the sequence of steps on the group's website: https://www.rediapp.org/index.php/en [62].

## Discussion

### Scale validation

Following the studies of Bohlmeijer et al. [49], Aguado et al. [63] and Cebolla et al. [48] on the validation of the original version of the FFMQ, a factorial model of correlation between the five dimensions of mindfulness has been proposed: observing, describing, acting with awareness, non judging internal experience and no-reactivity to internal experience, without a higher level of global measurement of mindfulness. The confirmatory factor analysis showed an adequate load of the items, but it was necessary to eliminate item 18 ("*When I have disturbing thoughts or images I am able to notice them without reacting*") of the dimension "non-reactivity", because it was not significant. The standardized factorial weights of the items are adequate, all greater than 0.30, being item 15 ("*I perceive the smell and smell of things*") the most influential in the dimension "observing", item 1 ("*I am good at finding the words to describe my feelings*") in the dimension "describing", item 22 ("*I do tasks automatically*, *without being aware of what I do*") in the dimension " acting with awareness", item 19 ("I think some of my emotions are bad or inappropriate and I should not feel them") in the dimension "non judging internal experience", and item 3 ("*I observe my feelings without getting lost in them*") in the dimension " non reactivity."

All the items, except for item 18, are correlated, and significantly influence their corresponding dimension, so that the FFMQ-SF questionnaire measures five different but related dimensions of mindfulness (all dimensions of the FFMQ-SF fit well to the data obtained in the sample studied). The non-significance of item 18 could have been due to a problem in the interpretation of the item, as in the study of Baer, Samuel and Lykins [64] on the original FFMQ scale, in which four items had an ambiguous interpretation. Also in the study of the psychometric properties of this scale in the Argentine population they demonstrated how the "non-reactivity" dimension (to which item 18 belongs) is not significant in the structure of mindfulness in people with little meditative experience [65]. There were five pairs of correlated errors with the Pearson coefficient higher than |0.3| that belonged to different factors. These problematic correlations occurred between items belonging to the "non-reactivity" dimension and other dimensions, which again maintain the thesis of no significance of this dimension within the structure of mindfulness in people with little meditative experience [64]. Another correlations between the error terms appeared, but with less strength ‒ 5 of 7 correlations implied the “observing” dimension, and three of them were correlations between “observing” and “acting with awareness”. As Baer and colleagues note [47], who observed the difference between groups of non-meditators and meditators on the scale of non-reactivity, the dimension of non-reactivity should be understood as a result of the continued practice of mindfulness. In the same way, it has been proposed that the “observe” facet should be excluded when using the sacle with people not experienced in the practice of mindfulness.

The results on the goodness of fit of the non-hierarchical, five-dimensional factorial model met the criteria for good and acceptable model adjustment (after eliminating item 18 and despite the correlations detected among the errors included in the model), explaining a large part of the variance, 55.5%. This improves the results obtained in the Japanese validation of the FFMQ of Sugiura et al. [66] which obtained an explained variance of 46%, or in the study by Baer et al. with 33% of the total variance explained [47].

With regard to the internal consistency of the dimensions, once item 18 was eliminated, the internal consistency of the "non-reactivity" dimension increased. The 5 dimensions presented an internal consistency of acceptable to good (values of Cronbach's alpha from 0.65 to 0.80). Values similar to those obtained by Bohlmeijer et al. in the validation of the Dutch short version[49], by Tran, Glück and Nader [50] in the validation of the German short version and by Hou et al. in the validation of Chinese short version [51].

In the present investigation, the "observe" dimension and the "non-reactivity" dimension present a Cronbach's alpha between questionable and acceptable. Aguado et al. [63] in the validation to the Spanish of the FFMQ questionnaire, in a non-clinical sample, reflected that the dimension "observe" only has correlations with meditators because of they ability to observe, without judgment or reactivity, which is functional and is related to the practice, and therefore the FFMQ should be interpreted with caution when used with non-meditative samples. It goes in line with the results of a recent clinical trial where the scale has been applied in both forms (long and short) of FFMQ [67] in 3 samples: a convenience sample of adults, experienced meditators, and a sample of patients with recurrent depression to whom the MBCT was to be administered, reaching a conclusion that the measurement of the five facets of mindfulness before and after the treatment should be done with the questionnaire with four factors, excluding the facet observation, while the post-MBCT should be done with the five-factor questionnaire.

This would justify this adjusted value in the internal consistency of the "observe" dimension of the FFMQ-SF questionnaire, validated in this study. The values below 0.70 in the dimension "non-reactivity" (made up of four items in the present research, instead of five, due to the elimination of item 18) have also been obtained in the questionnaire validations in other languages. In its Chinese version with a Cronbach's alpha of 0.64 [68], in its Norwegian version of 0.66 [69] and in its Japanese version of 0.67 [66].

These low values in the internal consistency of the dimension "non-reactivity" reveal a possible difficulty in the compression of the theoretical construct. Tran, Glück and Nader [50] detected in their adaptation of the FFMQ to the German, that the selection of the 24 items of the FFMQ-SF of Bohlmeijer et al. [49] may not be easily applicable in other languages or more heterogeneous samples. The effects of the language and the effects of the meditation experience on the adjustment of the model can produce alterations in the values of the internal consistency of the dimensions of the FFMQ-SF. Questionnaire FFMQ-SF presents good psychometric qualities of reliability and validity in its five component dimensions of mindfulness, being an adequate questionnaire for the evaluation of mindfulness in non-clinical multi- occupational population.

### Associations between five facet of mindfulness short form and depression symptoms

#### Practical implications of the study

Focusing on the relationship between the scores obtained in the different facets of the FFMQ-SF and presenting symptoms of depression now or in the past, it has been established that there is a high relationship between all facets of Mindfulness except in the facet of observing and suffering an active episode of depression. This fact supports the effectiveness of Mindfulness interventions in the treatment of depression, since these interventions, by increasing the capabilities of people with depression in these areas, would also modify their state of depression [70–72]. In relation to having suffered recurrences of depressive episodes, the facets that appeared as relevant were those of "acting with awareness" and "non-reactivity". The signfication obtained from the "acting with awareness" facet could be explained through the non-mechanization of their activities, the improvement in healthy living habits and health behaviors, while "non-reactivity" would be related to emotional regulation [71,73].

The practical implication of our results will allow adapting therapy for depression According to the results, the dimension that indicates more risk of suffering or suffering from depression is acting with awareness, obtaining patients with depression or with previous episodes with low results. Also the low result in the non- reactivity facet should be taken into account when designing a specific mindfulness- based intervention. Based on these results, and as a final conclusion, we highlight that working on these two facets is the key to preventing recurrent episodes of depression. As for the last conclusion, in a recent study about a recurrent depression treatment a factorial difference has been found between pre-MBCT and post-MBCT measurements [47,74]

### Limitations of the study

The main limitation of this study was the transversal design that did not allow to establish causal relationships between the variables. Another limitation is the use of scales and self-administered questionnaires that are mediated in their responses by social desirability, subjectivity, awareness, interpretation, motivation and the will of the participants, which could bias the results. Mindfulness assessment scales have been criticized because subjects may not be fully aware of their ability to experience the present moment [75].

The FFMQ presents the limitation of being sensitive to the meditative experience, with a more intense differential response effect when one is less familiar with the content of the item [76]. Disregarding the meditating experience of the participating subjects is another limitation, since it has been demonstrated that some dimensions of the scales used are sensitive to the fact of being a meditator
